# PAI-1 is a vascular cell–specific HIF-2–dependent angiogenic factor that promotes retinal neovascularization in diabetic patients

**DOI:** 10.1126/sciadv.abm1896

**Published:** 2022-03-02

**Authors:** Yaowu Qin, Jing Zhang, Savalan Babapoor-Farrokhran, Brooks Applewhite, Monika Deshpande, Haley Megarity, Miguel Flores-Bellver, Silvia Aparicio-Domingo, Tao Ma, Yuan Rui, Stephany Y. Tzeng, Jordan J. Green, M. Valeria Canto-Soler, Silvia Montaner, Akrit Sodhi

**Affiliations:** 1 Wilmer Eye Institute, Johns Hopkins University School of Medicine, Baltimore, MD 21287, USA.; 2 EENT Hospital, Fudan University, Shanghai 200031, China.; 3 State Key Laboratory of Ophthalmology, Zhongshan Ophthalmic Center, Sun Yat-Sen University, Guangzhou 510064, China; 4 *CellSight* Ocular Stem Cell and Regeneration Research Program, Department of Ophthalmology, Sue Anschutz-Rodgers Eye Center, University of Colorado School of Medicine, Aurora, CO 80045, USA.; 5 Department of Oncology and Diagnostic Sciences, Greenebaum Cancer Center, University of Maryland, Baltimore, MD 21201, USA.; 6 Department of Biomedical Engineering, Institute for NanoBioTechnology, and the Translational Tissue Engineering Center, Johns Hopkins University School of Medicine, Baltimore, MD 21231, USA.

## Abstract

For patients with proliferative diabetic retinopathy (PDR) who do not respond adequately to pan-retinal laser photocoagulation (PRP) or anti–vascular endothelial growth factor (VEGF) therapies, we hypothesized that vascular cells within neovascular tissue secrete autocrine/paracrine angiogenic factors that promote disease progression. To identify these factors, we performed multiplex ELISA angiogenesis arrays on aqueous fluid from PDR patients who responded inadequately to anti-VEGF therapy and/or PRP and identified plasminogen activator inhibitor-1 (PAI-1). PAI-1 expression was increased in vitreous biopsies and neovascular tissue from PDR eyes, limited to retinal vascular cells, regulated by the transcription factor hypoxia-inducible factor (HIF)-2α, and necessary and sufficient to stimulate angiogenesis. Using a pharmacologic inhibitor of HIF-2α (PT-2385) or nanoparticle-mediated RNA interference targeting *Pai1*, we demonstrate that the HIF-2α/PAI-1 axis is necessary for the development of retinal neovascularization in mice. These results suggest that targeting HIF-2α/PAI-1 will be an effective adjunct therapy for the treatment of PDR patients.

## Teaser

HIF-2α accumulation in hypoxic vascular cells promotes PAI-1 expression and retinal neovascularization in diabetic patients.

## INTRODUCTION

Sustained hyperglycemia leads to damage to the microvasculature (i.e., endothelial cells, pericytes, and smooth muscle cells) in diabetic patients (*1*). Clinically, this manifests in the retina as diabetic retinopathy (DR), the most common microvascular complication in the diabetic population and the leading cause of vision loss among working adults in the developed world (*2*). Significant sustained hyperglycemia can lead to sufficient damage to the retinal vasculature (*3*) to promote capillary dropout, leading to retinal ischemia and the secretion of the angiogenic mediators that promote neovascularization (NV) (*4*). The development of retinal NV in diabetic patients heralds the progression from nonproliferative diabetic retinopathy (NPDR) to proliferative diabetic retinopathy (PDR) (*5*). PDR remains the most difficult consequence of diabetic eye disease to treat and can result in vitreous hemorrhage, tractional retinal detachment, and/or neovascular glaucoma, which can lead to profound, often irreversible vision loss.

Until recently, the standard of care for PDR had been pan-retinal scatter laser photocoagulation (PRP), an inherently destructive procedure in which the peripheral ischemic retina is sacrificed (burned) with a laser to reduce the hypoxic drive promoting the expression of angiogenic mediators (*6*). Although effective in reducing the risk of central vision loss, PRP can result in decreased peripheral and night vision in treated patients. Moreover, some patients with PDR respond inadequately to PRP and can progress despite appropriate treatment. This has prompted investigation into new therapeutic options for the treatment of patients with PDR.

In this regard, recent clinical studies have demonstrated that monthly intraocular injections with therapies targeting a single hypoxia-driven angiogenic mediator, vascular endothelial growth factor (VEGF), can prevent or delay the development of retinal NV in many diabetic patients (*7*–*10*). Monthly injections with “anti-VEGF therapy” resulted in a reduction of progression from NPDR to PDR from 34% in the sham-treated group to just over 11% in diabetic patients treated with monthly anti-VEGF therapy (*8*, *9*). However, by year 3, the number of patients with NPDR treated with anti-VEGF therapy who progressed to PDR reached 18% (*8*, *9*), suggesting that therapies targeting VEGF alone may only delay DR progression in many diabetic patients. Moreover, in a subset of patients with mature florid NV, PDR can be remarkably resistant to treatment with anti-VEGF therapy. Collectively, these observations suggest that other angiogenic factors, in addition to VEGF, may contribute to the development, survival, and progression of retinal NV. The observation that some patients with PDR who undergo PRP also respond inadequately to treatment further demonstrates that preventing the release of other angiogenic mediators (including VEGF) by the ischemic retina is similarly not sufficient to promote the regression of retinal NV in all patients with PDR.

On the basis of these clinical observations, we hypothesized that vascular cells—endothelial cells and pericytes—within neovascular tissue may themselves secrete autocrine and/or paracrine factors in addition to VEGF that allow these pathological vessels to survive (or grow) despite current therapies. Vascular cells are not targeted by PRP, and the angiogenic mediators they secrete (other than VEGF) would also not be responsive to current anti-VEGF therapies. Here, we set out to identify novel vascular cell autonomous angiogenic factors that facilitate the development and proliferation of retinal NV in patients with PDR despite currently available therapies. We observed that plasminogen activator inhibitor-1 (PAI-1) expression is increased in the eyes of patients who respond inadequately to anti-VEGF therapy. PAI-1 expression was limited to endothelial cells and pericytes, regulated by the transcription factor hypoxia-inducible factor (HIF)-2α, and necessary and sufficient to stimulate angiogenesis. Inhibition of PAI-1 expression prevented the development of retinal NV in mice, suggesting that therapies targeting the HIF-2α/PAI-1 axis will be an effective adjunct approach for the treatment of patients with PDR.

## RESULTS

### Inhibition of late surge of HIF-2α expression is sufficient to block retinal NV in OIR mice

**Fig. 1.**
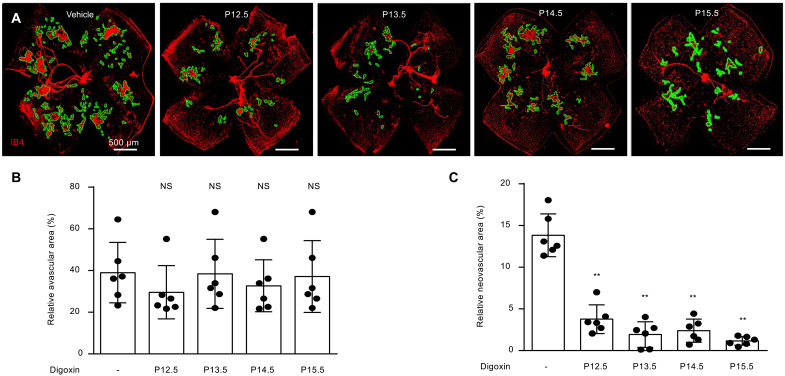
Inhibition of late surge of HIF-2α expression prevents retinal NV in OIR mice. (**A**) Retinal NV (outlined) in OIR mice at P17 following a single injection with digoxin (2 mg/kg) at P12.5, P13.5, P14.5, or P15.5, respectively. (**B** and **C**) Quantitation of avascular retina (B) and retinal NV (C) at P17 after treatment with digoxin in OIR mice. *n* = 6 to 8 animals. Data are shown as means ± SD. Statistical analyses were performed by one-way ANOVA with Bonferroni’s multiple-comparison test. *P < 0.05; **P < 0.01; NS, not significant.

We recently reported that the ischemic stage of the oxygen-induced retinopathy (OIR) model is characterized by a time-dependent accumulation of HIF-1α and HIF-2α in the ischemic inner retina (*11*). Specifically, we observed a transient accumulation of HIF-1α from postnatal day (P)12.5 (12 ½ days) to P13 followed by sustained accumulation of HIF-2α from P14 to P17. Expression of HIF-1α after P14 and expression of HIF-2α before P14 were not observed (*11*). HIF-2α accumulation was observed in two overlapping phases: transient accumulation in the inner nuclear layer (INL) from P14 to P15 and sustained accumulation within the ganglion cell layer (GCL) from P14 to P17 (*11*). Although this pattern may not reflect the expression of HIF-1α and HIF-2α in patients with ischemic retinopathies (*11*–*14*), it provides an opportunity to examine the contribution of HIF-2—and HIF-2–regulated angiogenic mediators—to the development of retinal NV. To this end, we treated OIR mice with a single intraperitoneal injection of the pharmacologic HIF inhibitor, digoxin (*15*), which results in a transient inhibition of HIF-1α and HIF-2α expression in OIR mice (*11*). Following a single injection with digoxin at P12.5, P13.5, P14.5, or P15.5, we observed a marked reduction in retinal NV in OIR mice at P17 (Fig. 1, A to C). Late inhibition of HIFs with digoxin at P15.5 was at least as effective as earlier inhibition of HIF with digoxin at P12.5, suggesting that the late, sustained expression of HIF-2α is critical for the growth and proliferation of retinal NV in OIR mice.

### Vascular cells in GCL express HIF-2α but not HIF-1α in OIR mice

**Fig. 2.**
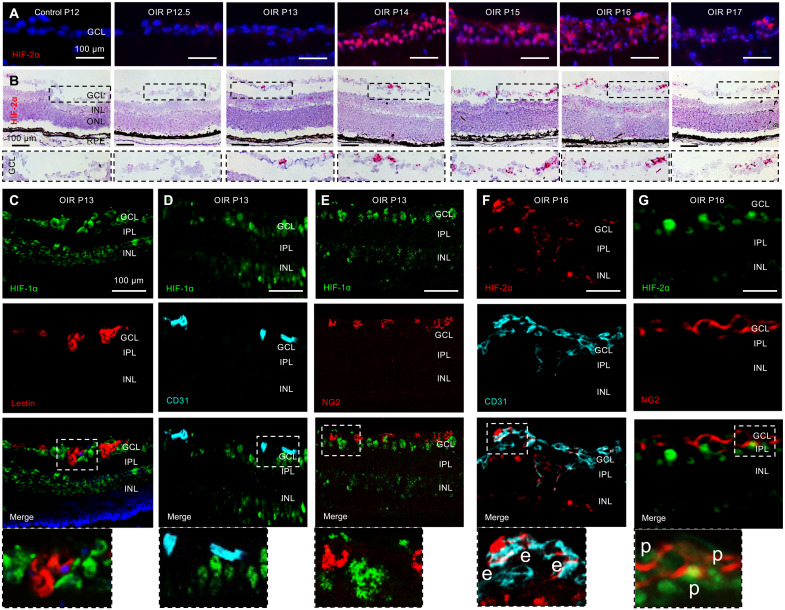
Vascular cells in NV tissue express HIF-2α but not HIF-1α in OIR mice. (**A**) Accumulation of HIF-2α in the GCL in OIR mice from P12 to P17. (**B**) Increased *Hif2α* mRNA expression in the GCL by RNAscope. (**C** to **E**) Coexpression of HIF-1α with isolectin B4 (lectin), CD31, or chondroitin sulfate proteoglycan 4 (NG2) was not detected in OIR mice retinas at P13 by immunofluorescence (IF). (**F** and **G**) Coexpression of HIF-2α with endothelial cell marker CD31 (e) or the pericyte marker NG2 (p) was observed in OIR mice retinas at P16. *n* = 4 to 6 animals; GCL, ganglion cell layer; IPL, inner plexiform layer; INL, inner nuclear layer; ONL, outer nuclear layer; RPE, retinal pigment epithelium. Scale bars, 100 μm.

Careful examination of the GCL in OIR mice demonstrated an increase in HIF-2α protein expression (Fig. 2A) that corresponded to an increase of *Hif2a* mRNA expression (Fig. 2B). This suggested a tightly regulated expression of both *Hif2a* mRNA and HIF-2α protein in the GCL following ischemic injury. While expression of HIF-1α was excluded from the retinal vessels (Fig. 2C) and coexpression of HIF-1α with the endothelial cell marker CD31 (Fig. 2D) or the pericyte marker NG2 (Fig. 2E) was not observed, increased expression of HIF-2α was observed within retinal neovascular tissue and was coexpressed with CD31 (Fig. 2F) and, to a lesser extent, the pericyte marker NG2 (Fig. 2G). Collectively, these results suggest that HIF-2α, but not HIF-1α, is expressed in neovascular tissue in ischemic retina, and expression of HIF-2α in these cells may be necessary for the development and/or progression of retinal NV in OIR mice.

### HIF-2–dependent angiogenic mediators promote retinal NV in vascular endothelial cells

**Fig. 3.**
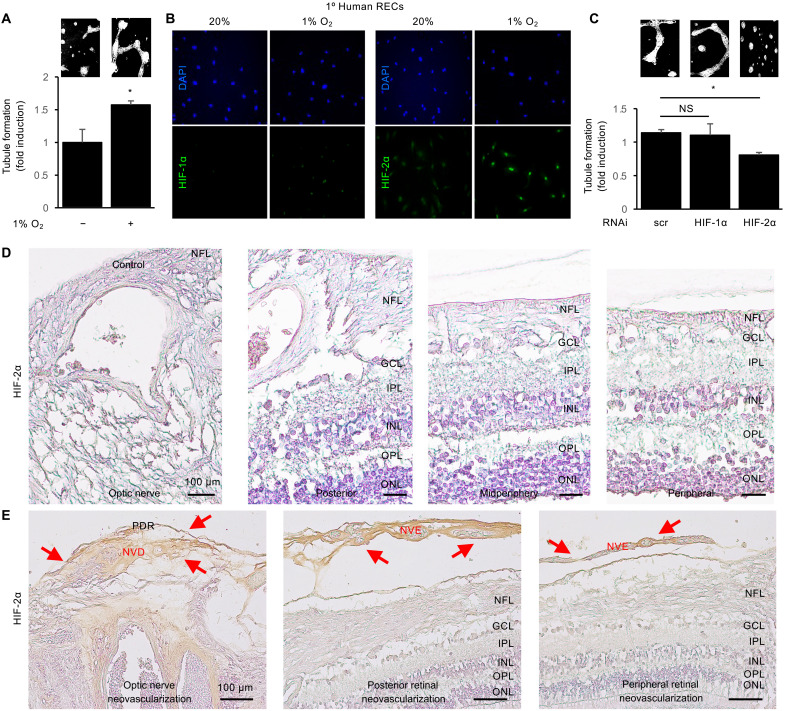
HIF-2–dependent angiogenic mediators promote retinal NV in PDR. (**A**) Tubule formation in primary human retinal endothelial cells (HRECs) treated with conditioned media from HRECs exposed to hypoxia (1% O_2_) or normoxia (20% O_2_). (**B**) Accumulation of HIF-1α (left) and HIF-2α (right) in HRECs cultured in hypoxia or normoxia by IF. (**C**) Tubule formation in HRECs exposed to hypoxia after knockdown of HIF-1α or HIF-2α by RNAi. (**D**) Expression of HIF-2α near the optic nerve, or in the posterior, mid-periphery, or periphery of autopsy eyes from nondiabetic control patients. (**E**) Expression of HIF-2α (red arrows) in neovascular tissue overlying the optic nerve (i.e., NV of the disc or NVD) and the retinal surface (i.e., NV elsewhere or NVE) in the optic nerve, posterior, or periphery of PDR eyes. NFL, nerve fiber layer; GCL, ganglion cell layer; IPL, inner plexiform layer; INL, inner nuclear layer; OPL, outer plexiform layer; ONL, outer nuclear layer. Data are shown as means ± SD. Statistical analyses were performed by two-tailed unpaired Student’s *t* test (A) or one-way ANOVA with Bonferroni’s multiple-comparison test (C). *P < 0.05. Scale bars, 100 μm.

To directly examine the contribution of HIF-2–dependent vascular cell–derived angiogenic mediators to the promotion of angiogenesis in PDR, we performed endothelial cell tubule formation assays on primary human retinal endothelial cells (HRECs) treated with their own conditioned media. We observed an increase in tubule formation in HRECs treated with conditioned media from HRECs cultured in hypoxia (1% O_2_) compared to conditioned media from HRECs cultured in normoxia (20% O_2_; Fig. 3A). Examination of HRECs treated with hypoxia demonstrated a modest increase in HIF-1α expression and a more marked increase in HIF-2α expression (Fig. 3B). Accordingly, knockdown of HIF-2α expression—but not HIF-1α expression—in hypoxic HRECs reduced the ability of their conditioned media to promote tubule formation (Fig. 3C).

To assess whether HIF-2 could play a similar role in neovascular tissue in patients with PDR, we examined HIF-2α expression in autopsy eyes from control (Fig. 3D) or PDR (Fig. 3E) patients; the patients with PDR had previously been treated with PRP. We did not observe significant expression of HIF-2α near the optic nerve or in the posterior, midperiphery, or periphery of control eyes (Fig. 3D). However, we observed marked expression of HIF-2α in neovascular tissue overlying the optic nerve (i.e., NV of the disc or NVD) and the retinal surface (i.e., NV elsewhere or NVE) in PDR eyes (Fig. 3E). Together, these observations support a role for HIF-2–regulated angiogenic mediators expressed by vascular cells in neovascular tissue in the promotion of retinal NV in PDR.

### PAI-1 expression is increased in PDR patients with low VEGF levels

We have previously observed that despite variable levels of VEGF, the aqueous fluid (AF) of diabetic patients with PDR is highly angiogenic compared to nondiabetic control patients (*12*). To identify vascular cell paracrine angiogenic mediators, we collected AF from a subset of patients with PDR who had been treated with PRP, or who had been treated with PRP and monthly anti-VEGF therapy, but did not respond adequately to treatment (i.e., patients who had active NV despite treatment and ultimately required vitrectomy surgery despite medical management). All the AF samples selected had high angiogenic potential (as measured by tubule formation assay) compared to samples from nondiabetic controls but had markedly lower VEGF levels than those in nondiabetic controls (table S1), consistent with their prior treatment with PRP and/or anti-VEGF therapy. While limiting the number of patient samples we could include in this analysis (four PDR patients with PRP, four PDR patients with PRP and anti-VEGF therapy, and four nondiabetic controls), these strict criteria enriched these samples for angiogenic mediators that were not dependent on elevated levels of VEGF. We then used a multiplex enzyme-linked immunosorbent assay (ELISA) angiogenesis array to examine the expression levels of angiogenic cytokines, growth factors, and inflammatory mediators. We observed a minimum twofold increase in the levels of three factors (PAI-1, Tissue inhibitor of metalloproteinases-1, and angiogenin) in four of four PDR patients who underwent PRP and had low levels of VEGF (PDR, low VEGF; table S1) compared to control patients (fig. S1A). Two additional factors [CXCL16 and insulin-like growth factor binding protein 2 (IGFBP2)] were elevated a minimum of twofold in three of four PDR, low VEGF patients compared to controls (fig. S1A). We next examined the aqueous from four patients with PDR who underwent PRP and had monthly treatment with anti-VEGF therapy (PDR, anti-VEGF), who also had low levels of measured VEGF (table S1) but had clinical evidence of an inadequate response to treatment (as described above). Among these, all five factors were elevated a minimum of twofold in three of four PDR, anti-VEGF patients (fig. S1B). Of these five factors, only PAI-1 and CXCL16 were increased by at least 2-fold in seven of eight samples, and only PAI-1 and IGFBP2 demonstrated a >3-fold mean increase in the two sets of patients with PDR (fig. S1, C and D).

### PAI-1 mRNA and protein expression are increased in retinal NV tissue in OIR mice

**Fig. 4.**
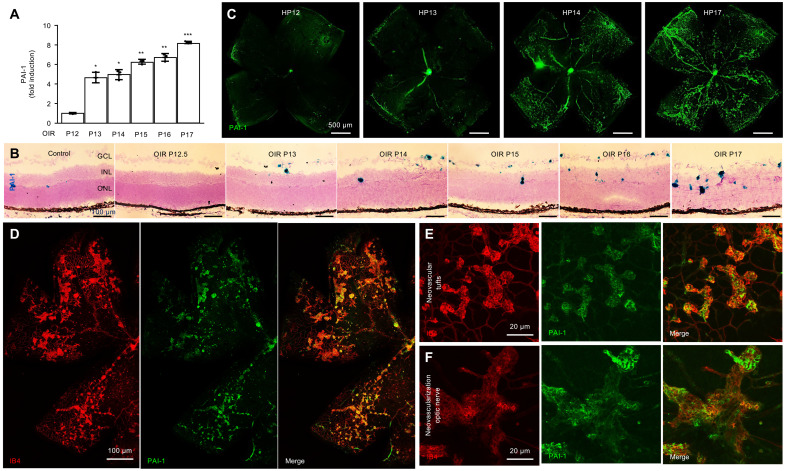
PAI-1 expression is increased in retinal NV tissue in OIR mice. (**A** and **B**) *Pai1* mRNA expression during the ischemic phase (P12 to P17) in OIR mice retinas by qPCR (A) and RNAscope (B). (**C**) PAI-1 protein expression during the ischemic phase in retinal flat mounts in OIR mice by IF. (**D**) Colabeling of PAI-1 with the vascular cell marker isolectin B4 (IB4) in retinal flat mounts in OIR mice by IF. (**E** and **F**) Colabeling of PAI-1 with IB4 in retinal flat mounts at the optic nerve (F) and overlying the retina (E) in OIR mice by IF. *n* = 6 to 8 animals; GCL, ganglion cell layer; INL, inner nuclear layer; ONL, outer nuclear layer. Data are shown as means ± SD. Statistical analyses were performed by one-way ANOVA with Bonferroni’s multiple-comparison test. *P < 0.05; **P < 0.01; ***P < 0.001. Scale bars, 500 μm (C), 100 μm (B and D), and 20 μm (E and F).

There is accumulating evidence for genetic susceptibility to the development of DR (*16*). Emerging evidence supports a link between polymorphisms in PAI-1 with an increased risk for diabetic nephropathy and DR (*17*). PAI-1 expression has been previously implicated in patients with diabetic eye disease (*18*). Nonetheless, the role of PAI-1 in the development and/or progression of PDR remains unclear. To examine the contribution of PAI-1 to the development of retinal NV in patients with PDR, we first examined the expression of PAI-1 in OIR mice. *Pai1* mRNA expression increased during the ischemic phase (P12 to P17) in OIR mice (Fig. 4, A and B). This correlated with an increase in PAI-1 protein expression in retinal flat mounts (Fig. 4C), in which expression of PAI-1 was noted to be highly expressed in areas of NV. Colabeling of PAI-1 with the vascular cell marker isolectin B4 (1,4-butanediol diacrylate) demonstrated significant expression of PAI-1 in neovascular tissue (Fig. 4D), including the neovascular tissue at both the optic nerve (NVD; Fig. 4E) and overlying the retina (NVE; Fig. 4F).

### PAI-1 expression is increased in patients with PDR

**Fig. 5.**
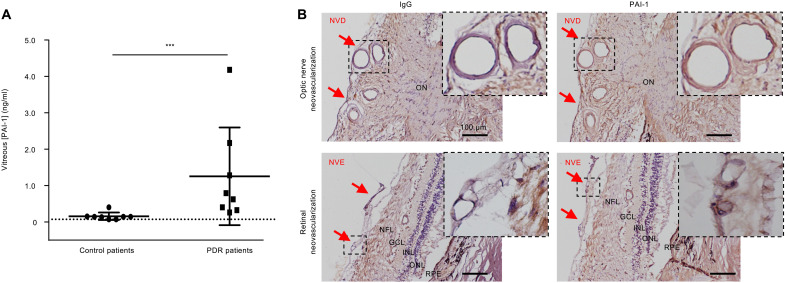
PAI-1 expression is increased in patients with PDR. (**A**) Expression of PAI-1 in vitreous samples from patients with PDR or nondiabetic control by ELISA. (**B**) Expression of PAI-1 in neovascular tissue from autopsy eyes from patients with PDR, including both NV at the optic nerve (NVD, red arrows) and over the ischemic retina (NVE, red arrows). IgG (immunoglobulin G) (left lane) was used as a negative control. NFL, nerve fiber layer; GCL, ganglion cell layer; INL, inner nuclear layer; ONL, outer nuclear layer; RPE, retinal pigment epithelium. ON, optic nerve; NVD, neovascularization of the disc; NVE, neovascularization elsewhere. Data are shown as means ± SD. Statistical analyses were performed by two-tailed unpaired Student’s *t* test. ***P < 0.001. Scale bars, 100 μm.

We next examined expression of PAI-1 by ELISA in vitreous samples from patients with PDR compared to nondiabetic control patients with no prior history of ischemic retinal disease. We observed a marked increase (7.9-fold) in the levels of PAI-1 in untreated PDR patients (no prior history of PRP or anti-VEGF therapy) compared to nondiabetic control patients (Fig. 5A). We next determined the expression of PAI-1 in neovascular tissue from patients with PDR. We observed expression of PAI-1 within neovascular tissue from autopsy eyes from patients with PDR (Fig. 5B). This included both NV at the optic nerve (NVD) and over the ischemic retina (NVE), similar to what we observed in OIR mice (Fig. 4, E and F).

### PAI-1 is expressed in vascular endothelial cells and pericytes in retinal NV tissue in OIR mice

**Fig. 6.**
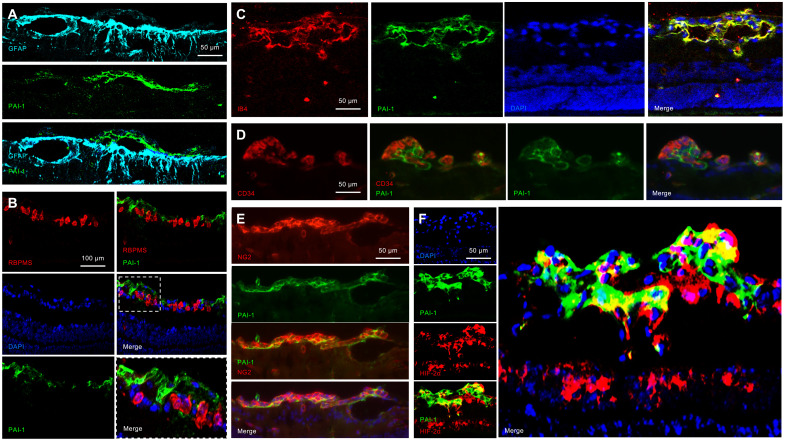
PAI-1 is expressed in vascular endothelial cells and pericytes in retinal NV tissue in OIR mice. (**A** and **B**) Retinal NV in the OIR model occurs at or above the GCL, containing resident glial cells (Müller cells, astrocytes, and microglia) and retinal ganglion cells (RGCs). Coexpression of PAI-1 with (A) GFAP (to label astrocytes and Müller cells) and (B) RBPMS (to label RGCs) was not detected. (**C** to **E**) Coexpression of PAI-1 with (C) IB4 (to label vascular and microglia cells), (D) CD34 (to label endothelial cells), and (E) NG2 (to label pericytes) was observed in retinal NV tissue in OIR mice by IF. (**F**) Coexpression of PAI-1 with HIF-2α in retinal NV tissue. *n* = 6 animals. Scale bars, 50 μm (A and C to F) and 100 μm (B).

We next set out to determine which cells in NV tissue express PAI-1. Neovascular fronds in the OIR model occur at or above the GCL, alongside resident glial cells and retinal ganglion cells (RGCs). Colabeling of PAI-1 with glial fibrillary acidic protein (GFAP) (to label astrocytes and Müller cells) demonstrated that PAI-1 was not expressed by glial cells (Fig. 6A). Similarly, colabeling of PAI-1 with the RGC marker RNA-binding protein with multiple splicing (RBPMS) demonstrated that PAI-1 was not expressed by RGCs (Fig. 6B). However, we did observe coexpression of PAI-1 and the vascular cell marker isolectin B4 (Fig. 6C). Colabeling of PAI-1 with CD34 demonstrated expression of PAI-1 in vascular endothelial cells (Fig. 6D). Similarly, colabeling of PAI-1 with NG2 demonstrated expression of PAI-1 in pericytes (Fig. 6E). Both endothelial cells and pericytes in NV tissue expressed HIF-2α (Fig. 2, F and G). Accordingly, coexpression of PAI-1 was observed in HIF-2α–expressing cells within NV tissue (Fig. 6F).

### PAI-1 is not expressed in human-induced pluripotent stem cell–derived three-dimensional retinal organoids lacking vascular cells

**Fig. 7.**
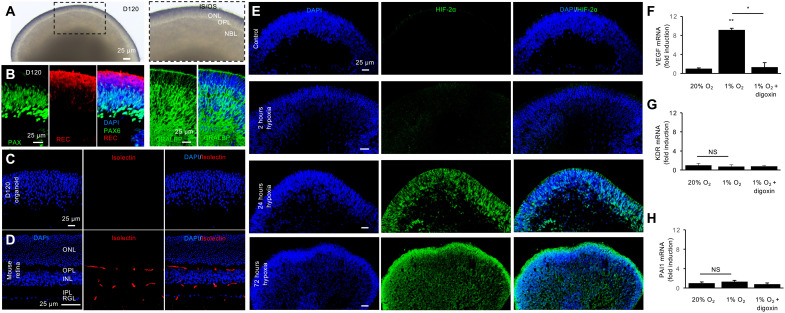
PAI-1 is not expressed in human-induced pluripotent stem cell–derived 3D retinal organoids. (**A** and **B**) D120 Human retinal organoids derived from hiPSCs containing mitotic retinal progenitors (expressing low levels of Pax6) and few newly differentiating bipolar cell precursors (lacking expression of Pax6 and recoverin, REC) within the neuroblastic layer, amacrine cells (expressing high levels of Pax6; most, if not all, ganglion cells have been lost by D120), outer retina photoreceptors (expressing recoverin, REC), and Müller glial cells (expressing CRALBP). (**C**) Vascular cells are not present in human retinal organoids as evidenced by the lack of staining with isolectin B4. (**D**) Cross section of mouse retina at P30 demonstrating expression of isolectin B4 in endothelial cells extending from the RGL across the INL toward the OPL, where the layer of retinal capillary networks normally ends. (**E**) Expression of HIF-2α over time in D120 human retinal organoids exposed to hypoxia for 2 hours to 3 days. Expression of *VEGF* (**F**), *KDR* (**G**), and *PAI1* (**H**) mRNA in human retinal organoids in normal conditions and after hypoxia treatment and inhibition by digoxin. NBL, neuroblastic layer; OPL, outer plexiform layer; ONL, outer nuclear layer; IS/OS, inner/outer segments. Data are shown as means ± SD. Statistical analyses were performed by one-way ANOVA with Bonferroni’s multiple-comparison test. *P < 0.05 and **P < 0.01. D120, 120-day retinoid culture. Scale bars, 25 μm.

To further characterize the cell specificity of PAI-1 expression in human retinal cells following hypoxic injury, we generated human-induced pluripotent stem cell (hiPSC)–derived three-dimensional (3D) retinal organoids. By 120 days (D120) of differentiation, the inner and outer retinal layers were clearly defined (Fig. 7A) and contained the precursors of the major retinal cell types (Fig. 7B), including outer retina photoreceptors (expressing recoverin), few newly differentiating bipolar cell precursors (lacking expression of recoverin and Pax6) within the neuroblastic layer, amacrine cells (expressing high levels of Pax6), and Müller cells [expressing the cellular retinaldehyde-binding protein (CRALBP)] (*19*). Notably absent in these 3D retinal organoids are retinal vascular cells, as evidenced by the lack of staining with isolectin B4 (Fig. 7C) compared to mouse retina (Fig. 7D). Culturing D120 retinal organoids in hypoxia (1% O_2_) resulted in robust expression of HIF-2α throughout the hypoxic retina (Fig. 7E). Increased expression of *VEGF* mRNA was observed in D120 retinal organoids exposed to hypoxia (Fig. 7F). This increase was blocked following treatment with digoxin, corroborating its HIF-dependent expression. Conversely, mRNA expression of the vascular endothelial cell gene, *KDR*, was not increased in D120 retinal organoids treated with hypoxia (Fig. 7G). Increased expression of *PAI1* mRNA was similarly not observed in hypoxia-treated D120 retinal organoids (Fig. 7H). Collectively, these results suggest that expression of PAI-1 in ischemic retina is limited to vascular cells.

### Expression of PAI-1 by vascular cells is dependent on HIF-2, not HIF-1

To further characterize the expression of PAI-1 by vascular cells, we first used an immortalized human umbilical vein endothelial cell line (iHUVEC). We have previously reported that hypoxic endothelial cells increase expression of matrix metalloproteinase 2 through both indirect (i.e., paracrine, VEGF-dependent) and direct (i.e., cell autonomous, HIF-dependent) mechanisms (*20*). To determine whether increased PAI-1 expression was a consequence of increased VEGF expression, we treated iHUVECs with increasing doses of recombinant human (rh)VEGF and did not observe an increase in *PAI1* mRNA expression (fig. S2A). Conversely, we did observe a digoxin-sensitive (i.e., HIF-dependent) increase in *PAI1* mRNA expression in iHUVECs treated with hypoxia (fig. S2B). Accordingly, use of pharmacologic hypoxia mimics CoCl_2_ and deferoxamine (DFO) as well as the prolyl hydroxylase inhibitors 1,4-dihydrophenonthrolin-4-one-3-carboxylic acid (1,4-DPCA) and dimethyloxalylglycine (DMOG) also increased the expression of *PAI1* mRNA in treated iHUVECs (fig. S2C). These results suggest an HIF-dependent regulation of PAI-1 in endothelial cells. We therefore examined the expression of *PAI1* mRNA in hypoxic iHUVECs following knockdown with HIF-1α, HIF-2α, or both, and observed a decrease in *PAI1* mRNA with knockdown of HIF-2α, but not HIF-1α (fig. S2D). Similar results were observed for *PAI1* mRNA in primary HRECs (fig. S2E) and in primary human pericytes (fig. S2F).

### PAI-1 promotes endothelial cell migration and angiogenesis

**Fig. 8.**
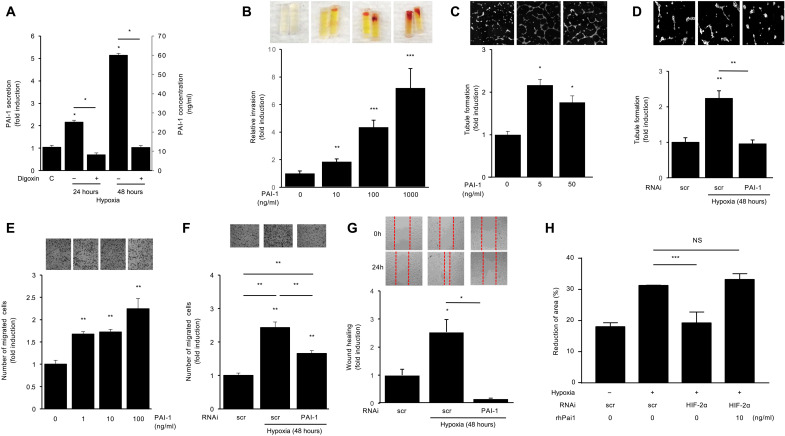
PAI-1 promotes endothelial cell migration and angiogenesis. (**A**) PAI-1 protein secretion in iHUVEC treated with digoxin in the presence and absence of hypoxia 1% O_2_ (24 to 48 hours). (**B**) PAI-1 (at indicated doses) promoted angiogenesis by using the directed in vivo angiogenesis assay (DIVAA). (**C**) Tubule formation in iHUVEC treated with increasing doses of PAI-1 reported as fold induction. (**D**) Tubule formation in iHUVEC treated with hypoxia after knockdown of PAI-1 by RNAi. (**E**) PAI-1 increased migration in iHUVEC by migration assay. (**F**) Knockdown of PAI-1 expression by RNAi in hypoxic iHUVECs reduced the ability of their conditioned media to promote migration. (**G**) Using wound healing assay to examine the iHUVEC migration exposed to hypoxia following the knockdown of PAI-1 by RNAi. (**H**) Using wound healing assay to examine the iHUVEC migration exposed to hypoxia following either the knockdown of HIF-2α by RNAi, treatment of recombinant human (rh)PAI-1, or both. Data are shown as means ± SD. Statistical analyses were performed by two-way ANOVA with Bonferroni’s multiple-comparison test (A) or one-way ANOVA with Bonferroni’s multiple-comparison test (B to H). *P < 0.05; **P < 0.01 and ***P < 0.001.

We next set out to determine whether PAI-1 could participate in the promotion of retinal NV. Conditioned media from iHUVECs express 20 to 60 ng/ml of PAI-1 protein after 24 to 48 hours of culture in hypoxia (Fig. 8A). To examine whether a similar concentration of PAI-1 could directly influence the growth of neovascular tissue in vivo, we used the directed in vivo angiogenesis assay (DIVAA). We observed a potent, dose-dependent increase in angiogenesis by PAI-1 compared to control in the DIVAA (Fig. 8B). These results were corroborated in vitro using the endothelial cell tubule formation assay in iHUVECs (Fig. 8C), similar to what was observed with conditioned media from hypoxic retinal endothelial cells (Fig. 3A). Accordingly, knockdown of PAI-1 expression by hypoxic iHUVECs prevented the ability of conditioned media from these cells to promote tubule formation (Fig. 8D).

It has previously been reported that PAI-1 promotes angiogenesis by stimulating endothelial cell migration (*21*). We therefore examined whether PAI-1 influenced endothelial cell migration and observed a dose-dependent increase in iHUVEC migration following treatment with PAI-1 (Fig. 8E). Knockdown of PAI-1 expression in hypoxic iHUVECs reduced the ability of their conditioned media to promote migration by iHUVECs (Fig. 8F). These results were corroborated in a second independent model of endothelial cell migration, the wound healing assay (Fig. 8G). Moreover, treatment of iHUVECs with rhPAI-1 was able to reverse the reduction of endothelial cell migration observed following knockdown of HIF-2α expression (Fig. 8H). Collectively, these results suggest that PAI-1 may be a key HIF-2–regulated angiogenic mediator elaborated by hypoxic vascular cells.

### Inhibition of HIF-2 blocks PAI-1 expression and retinal NV in OIR mice

**Fig. 9.**
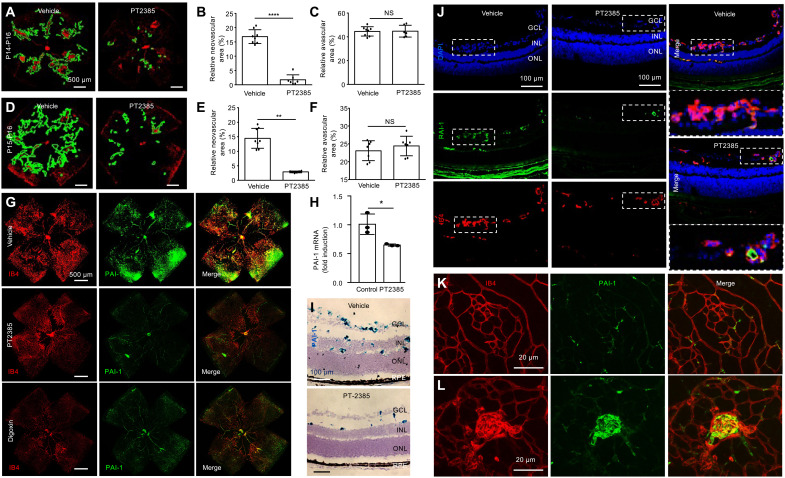
Inhibition of HIF-2α decreases PAI-1 expression in OIR mice. (**A** to **C**) Retinal NV (outlined) in OIR mice at P17 following daily oral lavage from P14 to P16 with the HIF-2α–specific pharmacologic inhibitor, PT2385 (30 mg/kg). (**D** to **F**) Retinal NV (outlined) in OIR mice at P17 following daily oral lavage from P15 to P16 with PT2385 (30 mg/kg). (**G**) IF colabeling of PAI-1 with IB4 in retinal flat mounts in OIR mice treated daily with either digoxin (0.5 mg/kg) or PT2385 (30 mg/kg). (**H** and **I**) *PAI1* mRNA expression in OIR mice treated with PT2385 by qPCR (H) and RNAscope (I). (**J**) Colabeling of PAI-1 with IB4 in cross section of OIR mice treated with PT2385 by IF. (**K** and **L**) Representative retinal flat mounts demonstrating the lack of PAI-1 in the absence of neovascular tissue (K), but presence of PAI-1 expression within the few residual neovascular tufts (L) in OIR mice treated with PT2385. *n* = 6 to 8 animals. GCL, ganglion cell layer; INL, inner nuclear layer; ONL, outer nuclear layer. Data are shown as means ± SD. Statistical analyses were performed by two-tailed unpaired Student’s *t* test. *P < 0.05; **P < 0.01; ****P < 0.0001; IF, immunofluorescence. Scale bars, 500 μm (A, D, and G), 100 μm (I and J), and 20 μm (K and L).

To specifically target HIF-2 function within the neovascular tissue, we next treated OIR mice with an HIF-2–specific pharmacologic inhibitor PT2385 (*22*). PT2385 selectively binds HIF-2α (*K*_d_ < 50 nM)—but not HIF-1α—to prevent it from binding to HIF-1β and has shown promise in preclinical studies (*22*) and clinical trials on patients with renal cell carcinoma (*23*, *24*). Treatment of OIR mice with PT2385 with daily oral lavage from P14 to P16 resulted in a marked decrease in retinal NV in OIR mice at P17 (Fig. 9, A to C). Similar results were observed following HIF-2 inhibition at P15 and P16 (Fig. 9, D to F), when HIF-2α expression is no longer detectable in the ischemic inner retina of OIR mice (*11*). To determine whether PAI-1 expression in OIR mice was strictly dependent on HIF-2, we treated OIR mice with PT2385 and examined expression of PAI-1. We observed a marked reduction in PAI-1 expression in animals treated with PT2385, similar to what we observed with the HIF-1 and HIF-2 inhibitor, digoxin (Fig. 9G). This correlated with a reduction in *Pai1* mRNA expression (Fig. 9, H and I). Expression of PAI-1 protein in OIR animals treated with PT2385 was limited to inner retinal vessels and the few residual neovascular tufts (Fig. 9J). In the absence of neovascular tissue, we did not observe PAI-1 expression in OIR mice treated with PT2385 (Fig. 9K). However, within the few residual neovascular tufts in OIR mice treated with PT2385, we observed strong expression of PAI-1 (Fig. 9L), suggesting that PAI-1 expression may be required for the growth of retinal NV.

### PAI-1 is necessary for pathological angiogenesis in OIR mice

We next set out to determine whether therapies specifically targeting PAI-1 could be an effective approach for the treatment of retinal NV. As postnatal retinal vascular development (P1 to P15) overlaps with both the hyperoxic (P7 to P12) and ischemic (P12 to P17) stages of the OIR model, it can be challenging to unravel the contribution of genes to developmental versus pathological angiogenesis using the OIR model with knockout mice. To overcome this obstacle, we developed a nanoparticle-based RNA interference (RNAi) approach to specifically knock down expression of genes in vivo by generating small interfering RNA (siRNA)–encapsulating nanoparticles using linear bioreducible poly(beta-amino ester)s (PBAEs) (fig. S3) (*25*). These biodegradable nanoparticles are designed to release siRNA cargo in an environmentally triggered manner upon cleavage of disulfide bonds in the polymer backbone in the reducing cytosolic environment.

**Fig. 10.**
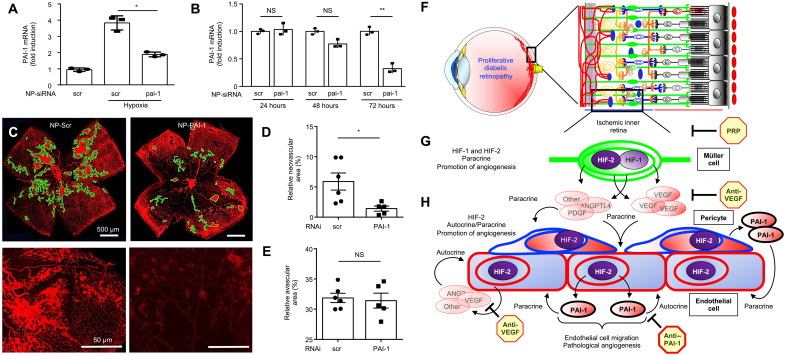
PAI-1 is necessary for pathological angiogenesis in OIR mice. (**A** and **B**) Expression of *PAI1* mRNA in iHUVEC exposed to hypoxia in vitro (A) and in OIR mice retinas in vivo (B) after treatment with polymer nanoparticle–mediated RNAi (NP-siRNA) knockdown of PAI-1. (**C**) Retinal NV (outlined) at P17 in OIR mice following the intravitreal injection with NP-pai-1 or NP-scr (as a control). (**D** and **E**) Quantitation of avascular retina and retinal NV at P17 after the intravitreal injection with NP-pai-1 or NP-scr. *n* = 6 to 8 animals. Data are shown as means ± SD. Statistical analyses were performed by one-way ANOVA with Bonferroni’s multiple-comparison test (A), two-way ANOVA with Bonferroni’s multiple-comparison test (B), or two-tailed unpaired Student’s *t* test (D and E). *P < 0.05; **P < 0.01. Scale bars, 500 μm. (**F** and **G**) A schematic representation of therapies for retinal NV in ischemic retinal disease. In ischemic retinopathies, decreased perfusion of the inner retina (F) results in accumulation of HIF-1α and HIF-2α in hypoxic retinal Müller cells (G), resulting in the secretion of angiogenic mediators. These paracrine secretions are effectively targeted by PRP. (**H**) While anti-VEGF therapy can inhibit VEGF released by endothelial cells and pericytes, other angiogenic autocrine/paracrine mediators released by retinal vascular cells are not effectively treated with PRP or anti-VEGF therapy. Therapies targeting the HIF-2–dependent expression of PAI-1 by retinal vascular cells may be an effective adjunct therapy for the treatment of ischemia-driven retinal NV.

We next used the nanoparticles to encapsulate *Pai1* siRNA (NP-PAI1). We observed efficient knockdown of *Pai1* mRNA expression in vitro and in vivo (Fig. 10, A and B). Intravitreal injection with NP-Scrambled (Scr) at P12 reduced the levels of NV compared to untreated mice, consistent with a prior report demonstrating the ability of RNAi to reduce retinal NV independent of its target (*26*). However, following a single intravitreal injection at P15, we observed a further inhibition of retinal NV with NP-PAI1 (Fig. 10, C and D) without influencing the area of avascular retina (Fig. 10E) compared to NP-Scr. These results suggest that therapies targeting the HIF-2–dependent expression of PAI-1 could be an effective adjunct approach in combination with PRP or anti-VEGF therapy for the treatment of retinal NV in patients with PDR (Fig. 10F).

## DISCUSSION

The anticipated rise in the global prevalence of diabetes will undoubtedly result in a concurrent increase in the number of patients with PDR, the leading cause of severe vision loss in diabetic patients (*2*, *27*). This will disproportionately affect underdeveloped countries where access to the resources necessary to diagnose and treat diabetes is already scarce. Current treatment options for PDR include scatter laser photocoagulation (i.e., PRP) and anti-VEGF therapy. While effective in quenching the expression of paracrine angiogenic mediators (e.g., VEGF) secreted by ischemic inner retinal cells (e.g., retinal Müller glial cells), PRP does not directly target NV; vascular cells within neovascular tissue can therefore continue to express angiogenic mediators (including VEGF) that can promote the progression of PDR.

An alternative approach for the treatment of PDR is monthly injections with anti-VEGF therapy. One explanation for the efficacy of anti-VEGF therapy may be that it targets VEGF secretion from both the ischemic inner retina and from neovascular tissue. Post hoc analyses from multicentered clinical trials using anti-VEGF therapy to treat diabetic macular edema have further demonstrated a significant reduction in the progression to PDR in some—but not all—patients with NPDR receiving monthly anti-VEGF therapy (*7*–*9*). These results were subsequently corroborated in prospective clinical trials (*10*). This suggests that other angiogenic mediator(s), in addition to VEGF, participate in the development and progression of PDR in diabetic patients.

VEGF plays a critical physiologic role promoting the health of the choriocapillaris, the vascular bed that supplies retinal photoreceptors (*28*–*31*). VEGF may also play a direct role as a neurotrophic factor for the neurosensory retina (*32*). These observations have raised concerns about the long-term consequences of sustained VEGF inhibition in the eye (*33*, *34*). Post hoc analyses of clinical studies assessing the treatment of patients with anti-VEGF therapy implicate chronic VEGF suppression in the development of retinal atrophy (*35*) and glaucoma (*36*). Collectively, these findings emphasize the importance of identifying additional therapeutic targets for treatment of diabetic eye disease.

In this regard, HIFs activate the transcription of multiple genes encoding angiogenic mediators that promote retinal NV in PDR, including VEGF. We have recently reported sustained expression of both HIF-1α and HIF-2α in patients with ischemic retinopathies (*11*). While both HIF-1 and HIF-2 cooperate in hypoxic Müller cells to promote VEGF expression, only HIF-1 was required to promote the expression of a second angiogenic mediator, ANGPTL4 (*11*). Accordingly, overexpression of a normoxia-stable mutant of HIF-1α was sufficient to promote NV in the mouse retina (*11*). This suggested that therapies targeting both HIF-1 or HIF-2 will be necessary to inhibit the expression of HIF-regulated angiogenic mediators by ischemic Müller cells. However, using both the OIR model and adult mouse retinal explants, we further observed that ischemic injury resulted in the staggered transient expression of HIF-1α and HIF-2α in the inner retina of mice. Consequently, targeting either HIF-1α or HIF-2α expression in inner retinal cells was sufficient to prevent retinal NV in OIR mice (*11*). These results help explain prior studies demonstrating that genetically targeting expression of either HIF-1α (*37*, *38*) or HIF-2α (*39*) effectively suppressed retinal NV in OIR mice.

Here, we extend studies on the role of HIF-regulated angiogenic mediators in ischemic retinopathies to the autocrine/paracrine secretions of vascular cells within neovascular tufts. Unlike ischemic Müller cells, vascular endothelial cells and pericytes within neovascular tufts are not typically targeted by PRP. Retinal vessels are purposely avoided when scatter laser is placed (to avoid an iatrogenic vascular occlusion). In addition, neovascular tissue is typically not amenable to laser uptake. Consequently, we hypothesized that paracrine secretions from hypoxic vascular cells within poorly perfused retinal vessels or neovascular tissue can continue to produce autocrine/paracrine angiogenic mediators, which can maintain (and, theoretically, promote) the progression of PDR despite PRP. While it has previously been reported that expression of HIF-1α in retinal endothelial cells can influence their angiogenic potential by regulating the expression of microRNAs (*40*), we demonstrate here that vascular cells within neovascular tissue in OIR mice express HIF-2α—but not HIF-1α—in the late ischemic stage in OIR mice. We further demonstrate that targeting HIF-2 within the vascular cells is sufficient to prevent the growth of retinal NV in OIR mice, suggesting that targeting HIF-2 may be an effective supplemental therapy for PDR.

We subsequently set out to identify specific HIF-2–dependent vascular cell–specific angiogenic mediators. To this end, we took advantage of our previous observation that AF from diabetic patients contains angiogenic mediators, in addition to VEGF, which participate in the promotion of PDR (*12*, *41*). We examined AF samples from PDR patients who responded inadequately to PRP and/or anti-VEGF therapy, had low levels of VEGF, but retained high angiogenic potential. Using an angiogenic array, we identified several potential angiogenic mediators that could contribute to the progression of PDR. While we ultimately focused on PAI-1 in the current study, we also identified two other proteins, CXCL16 and IGFBP2, in our screen for angiogenic mediators in aqueous samples from PDR patients who responded inadequately to current therapies. CXCL16 plays an important role in leukocyte recruitment and inflammation (*42*) and has been implicated in autoimmune and inflammatory diseases, including diabetes. While initial studies on CXCL16 in diabetes focused on diabetic nephropathy (*43*–*48*), recent work has implicated a role for CXCL16 in DR (*49*). HIF-dependent expression of CXCL16 and its endothelial cell receptor CXCR6 have previously been reported in response to hypoxia (*50*, *51*), and expression of CXCL16 has been reported to stimulate angiogenesis in cancer (*52*). Increased expression of IGFBP2 in the eyes of patients with ischemic retinal disease, including PDR (*53*–*55*), has also previously been reported. Both HIF-1 and HIF-2 have been reported to regulate expression of IGFBP2 (*56*, *57*), which has also been implicated in the promotion of angiogenesis (*58*, *59*). Future studies examining the contribution of CXCL16 and IGFBP2 will be necessary to determine whether they may also serve as effective targets for the treatment of PDR.

Here, we focused on the contribution of PAI-1 to the promotion of retinal NV in PDR. Accumulating evidence supports a link between polymorphisms in PAI-1 with an increased risk for DR (*17*). PAI-1 plays a central role in the regulation of plasmin formation by inhibiting plasminogen activators (i.e., urokinase-plasminogen activator and tissue type plasminogen activator) and has been implicated in pathological angiogenesis in cancer (*60*) and in the eye (*61*). Emerging evidence suggests that PAI-1 may contribute to the pathophysiology of DR (*18*). Nonetheless, how (and whether) PAI-1 contributes to the development of retinal NV in patients with PDR is not clear.

We report here that PAI-1 is expressed by neovascular tissue in OIR mice and in the eyes of patients with PDR. We further demonstrate that PAI-1 is specifically expressed by vascular endothelial cells and pericytes and that its expression requires HIF-2 but not HIF-1. We provide evidence linking PAI-1 to the development of pathological angiogenesis through its promotion of endothelial cell migration. Expression of PAI-1 by vascular cells was sufficient to promote endothelial cell tubule formation in vitro and pathological angiogenesis in mice. On the basis of its ability to promote endothelial cell migration, we predict that PAI-1 may mediate its effects by regulating the activity of small G proteins. However, additional studies will be necessary to examine the mechanism whereby PAI-1 contributes to endothelial cell migration and whether it requires PAI-1 enzymatic activity and/or whether urokinase or other proteases or integrins play a role.

Using a pharmacologic inhibitor of HIF-2, PT2385 (*22*), which has shown promise for the treatment of patients with renal cell carcinoma (*23*, *24*), we demonstrate that preventing HIF-2–dependent PAI-1 expression in retinal vascular cells prevents the development of retinal NV in OIR mice. In light of these findings, the recent FDA (Food and Drug Administration) approval of the sister drug, belzutifan, for cancers associated with von Hippel–Lindau disease (*62*) raises hope that this HIF-2 inhibitor could prove efficacious for PDR patients with retinal NV. Using a nanoparticle-based RNAi approach, we further demonstrate that targeting PAI-1 alone prevents the development of retinal NV in OIR mice. Collectively, our results suggest that therapies targeting the HIF-2/PAI-1 axis could become an important and effective tool in the arsenal of therapies for the treatment of recalcitrant NV in patients with PDR (Fig. 10, F and G).

## MATERIALS AND METHODS

### Reagents

rhPAI-1 ELISA kits were obtained from R&D Systems (no. DSE100). rhVEGF, PT2385 (no. HY-12867), digoxin (Chemical Abstracts Service no. 20830-75-5), and DFO (no. 138-14-7) were obtained from MedChemExpress. 1,4-DPCA and DMOG were obtained from Cayman Pharmaceuticals.

### Human cell culture

iHUVECs, primary HRECs, and primary human pericytes were obtained from Lonza and cultured according to the manufacturer’s protocols. All cells were cultured in high-glucose Dulbecco’s modified Eagle’s medium (DMEM) with 10% (v/v) fetal bovine serum (FBS) (Quality Biological) and 1% penicillin-streptomycin (Cellgro). Hypoxia chambers were used to expose cells and retinal organoids to 1% O_2_.

### Mice

Eight-week-old, pathogen-free female C57BL/6 mice (the Jackson Laboratory) and timed pregnant C57BL/6 mice (Embryonic day 14 gestation) (Charles River Laboratories) were treated in accordance with the Association for Research in Vision and Ophthalmology Statement for the Use of Animals in Ophthalmic and Vision Research and the guidelines of the Johns Hopkins University Animal Care and Use Committee.

### Retinal organoids

An hiPSC line derived from CD34^+^ cord blood was used here (A18945, Thermo Fisher Scientific) (*63*). Undifferentiated hiPSCs and derived retinal organoids were routinely tested for mycoplasma contamination by polymerase chain reaction (PCR). Cell culture, retinal differentiation, and organoid formation were conducted as previously described (*19*). Retinal organoids at 120 days of differentiation were used for experiments. Human retinal organoids were exposed to hypoxia for 2, 24, and 72 hours.

### OIR model

OIR experiments were performed as previously described (*64*). Briefly, C57BL/6 mice were placed in 75% O_2_ at P7. On P12, the mice were returned to room air and administered digoxin (0.5 or 2 mg/kg) by intraperitoneal injection and PT2385 (30 mg/kg) by oral lavage. Mice with body weight lower than 6 g at P17 were excluded from analysis. The data were collected from both males and females and the results were combined, as there was no apparent difference between sexes. The mice were euthanized at P17 for flat mounts. Mice retinal flat mounts were stained with GS-lectin B4 for quantitation of avascular retinal area and retinal NV as previously described (*65*). In brief, avascular retina and retinal NV were outlined using Image-Pro Plus (Count and Measure program). Area of avascular retina or total NV were then compared to the total retinal area. Two people independently performed the analyses.

Representative images for selected time points from a minimum of three independent experiments are shown. Data from four to eight pups were obtained at each time point.

### ELISA

Levels of secreted PAI-1 were measured in conditioned media from iHUVEC and vitreous from patients using human PAI-1 ELISA kits (R&D System) according to the manufacturer’s recommendations. Vitreous was diluted 1:10 for the PAI-1 ELISAs. ELISAs are representative of at least three independent experiments.

### siRNA studies

HIF-1α (ID no. 158953), HIF-2α (ID no. 158401), and PAI-1 (ID nos. 150176 and S10013) siRNA and a nontargeting control were purchased from Ambion. For in vitro knockdown experiments, cells were transfected with Lipofectamine 2000. The efficiency of siRNA was confirmed by immunoblot and/or reverse transcription and quantitative real-time PCR (qPCR) assays.

### In situ hybridization

RNA in situ hybridization was performed with RNAscope 2.5 HD Duplex Detection Reagent Kit (no. 323350, ACD) following the manufacturer’s protocol. Fresh eyecups, without prior fixation, were embedded into optimal cutting temperature (O.C.T.) compound (Tissue-Tek) and immediately frozen by liquid nitrogen. The frozen blocks were sectioned at a thickness of 14 μm and were assayed for *Pai1*mRNA (probe no. 18165A) and *Hif2a* mRNA (probe no. 18218C) versus negative control (probe no. 320751). The signal was visualized and captured by a Zeiss confocal microscope meta 710 laser scanning microscope (LSM) (Carl Zeiss Inc.).

### Immunohistochemistry

Details for antibodies are provided in table S2. Immunohistochemical (IHC) detection was performed with ABC system (Dako, Santa Clara, CA) according to the manufacturer’s protocol on ischemic retina from patients with PDR (obtained from the Wilmer Eye Institute Ocular Pathology Archives with approval from the Johns Hopkins School of Medicine Internal Review Board) as previously described (*66*, *67*). Detection of HIF-2α (no. NB-100-122, Novus Biologic) and PAI-1 (SC8979, Santa Cruz Biotechnology) by IHC was performed on cryopreserved human diabetic retina sections using a nitroblue tetrazolium development system using streptavidin alkaline phosphatase. Images were captured by scanning slides with the Aperio ScanScope program on Aperio ScanScope XT System (Leica Biosystems, Wetzlar, Germany).

### Immunofluorescence

Details for antibodies are provided in table S2. Immunofluorescence in primary hRECs were performed as previously described (*12*, *14*, *20*). Human retinal organoids were fixed in 4% paraformaldehyde for 1 hour, washed in phosphate-buffered saline (PBS) (2 × 5 min), and cryoprotected with a sucrose gradient (6.75, 12.5, and 25%, overnight at 4°C each) with a final incubation in 25% sucrose/OCT (2:1 ratio respectively) for 1 hour at room temperature (RT). Samples were embedded in 25% sucrose/OCT Tissue-Tek (Sakura), frozen, and stored at −80°C until used. Cryosections of 12- to 16-μm thickness were obtained and collected on Superfrost Plus slides. Sections were air-dried for 1 hour, washed in PBS (3 × 5 min), blocked in 10% goat serum in PBS with 0.25% Triton X-100 for 1 hour at RT, and incubated overnight with a primary antibody in 2% goat serum in PBS with 0.05% Triton X-100 at 4°C. The next day, the slides were washed in PBS (3 × 5 min) and incubated with an Alexa Fluor–conjugated secondary antibody (1:500; Molecular Probes) in PBS for 1 hour in the dark at RT. The slides were then washed in PBS (3 × 5 min), incubated in 4′,6-diamidino-2-phenylindole (DAPI) (1:1000 in PBS) for 10 min, and cover-slipped using DAKO fluorescent mounting medium. Similar procedures were applied for double and triple immunostaining. Fluorescence images were acquired with a Nikon C2 laser scanning confocal microscope (Melville, NY, USA). The images were minimally processed using Adobe Photoshop CS5 (San Diego, CA, USA).

The immunofluorescence for the OIR tissue (cross sections) was performed on cryopreserved retina tissue sections as previously described (*12*, *14*, *20*). Briefly, isolated retinas were fixed in 4% paraformaldehyde for 2 hours. After washing in buffered saline, the tissues were embedded in OCT containing 6% agarose (w/v), and 10-μm-thick slices were cut. Subsequently, the slices were incubated in a mixture of primary antibodies overnight at 4°C. After washing, the slices were incubated in secondary antibodies for 1 hour at RT. The slides were then incubated in DAPI (1:3000 in PBS) for 30 min and cover-slipped using DAKO fluorescent mounting medium.

For quantitation of retinal NV in mice, animals were sacrificed at the designated time points using CO_2_ asphyxiation, and eyes of mice were enucleated and fixed with a solution of 4% paraformaldehyde in PBS (Thermo Fisher Scientific) for 1 hour at RT, followed by washing with PBS for 10 min in a shaker. Retinas were then isolated and incubated in 0.5% bovine serum albumin solution overnight at 4°C. Retinas were washed with PBS three times for 10 min in the shaker and then stained with isolectin B4 (1:200 dilution in PBS; Invitrogen) overnight in 4°C. After washing three times for 10 min each in the shaker, the retinas were mounted. Images were captured by the LSM 710 confocal microscope (Carl Zeiss Inc., Thornwood, NY) or the EVOS FL Auto 2 Imaging System (Thermo Fisher Scientific, Waltham, MA).

### Reverse transcription and qPCR

Details for primers are listed in table S3. Total RNA was isolated from culture cells or retinas with PureLink RNA Mini Kit (no. 12183025, Invitrogen), and cDNA was prepared with reverse transcription kit (no. 205311, QIAGEN). qPCR was performed with PowerUp SYBR Green PCR Master Mix (no. A25742, Applied Biosystems) and MyiQ Real-Time PCR Detection System (Bio-Rad). Normalization was done using cyclophilin A for mouse tissue and cell lines and β-actin for human cell lines.

### Tubule formation assay

The tubule formation assay was performed as previously described (*12*, *68*). In brief, 50 ml of Matrigel was added to each well in 96-well plates for 30 min at 37°C. HRECs were seeded on Matrigel at 25,000 cells per well in 96-well plates in depleted FBS medium after treating with or without knockdown of HIF-1α or HIF-2α by RNAi. The cells were cultured 24 hours in a 1% O_2_ or 20% O_2_ incubator. Tubule formation was quantified using Angiogenesis Analyzer for ImageJ. Four fields were analyzed in each experiment, and each experiment was repeated three times.

### Endothelial cell migration assay

Endothelial cell migration was evaluated in a modified Boyden chamber assay using Transwell filters inserts with 8-μm pore size (Neuro Probe Inc.), as previously described (*69*). Briefly, iHUVECs were seeded into the upper chamber after treatment with or without knockdown PAI-1 by RNAi. Cells were grown in DMEM for 12 hours, and then cells were treated with or without PAI-1 (1, 10, or 100 ng/ml) for 24 hours. The number of migrated iHUVECs was analyzed under a microscope.

### Wound healing assay

iHUVECs were cultured in a six-well plate at a density of 1 × 10^4^ cells per well in growth medium. After the cells reached 80 to 90% confluence in each well, knockdown of PAI-1 expression by RNAi, scratches were made using a sterile tip. The monolayer of iHUVECs was incubated with a migration assay buffer consisting of serum-free medium. Images were captured at 0 and 24 hours. The wound healing area was analyzed using ImageJ software.

### Directed in vivo angiogenesis assay

The DIVAA was performed according to the manufacturer’s protocol (Trevigen). Briefly, 6-week-old female nude mice were anesthetized. A 1-cm incision was made on both dorsal-lateral surfaces of the animal, and two basement membrane extract (BME) premixed angioreactors were implanted on each side. Each angioreactor contained a total of 25 μl of BME premixed with heparin (20 μg/ml) and control medium or medium containing recombinant protein. Angioreactors were removed 11 days after implantation. The matrigel was digested with 300 μl of CellSperse solution for 1 hour (37°C), and endothelial cells were labeled with fluorescein isothiocyanate–lectin. Fluorescence was measured in 96-well plates (excitation 485 nm, emission 510 nm). Values for cell invasion are calculated as the ratio of relative fluorescent units of the test sample with respect to control sample.

### Multiplex ELISA angiogenesis arrays

To examine the expression levels of angiogenic mediators, we used R&D Systems’ Proteome Profiler Human Angiogenesis Array Kit (catalog no. ARY007). The assay was performed according to the manufacturer’s protocol. Briefly, 15 μl of reconstituted detection antibody cocktail was added to each prepared vitreous sample and incubated for 1 hour at RT. Then, this mixture was added to the wells of the four-well multidish and incubated at 4°C overnight on a shaker. The diluted streptavidin–horseradish peroxidase was incubated for 30 min at RT, and the membranes were exposed to x-ray film for 1 to 10 min. Pixel densities on the developed x-ray film were collected and analyzed using a transmission mode scanner and image analysis software.

### Nanoparticles

Bioreducible monomer 2,2-disulfanediylbis(ethane-2,1-diyl) diacrylate (BR6) was synthesized as previously described (*70*). Diacrylate monomer B4, side-chain monomer 4-amino-1-butanol (S4), and end-cap 2-(3-aminopropylamino)ethanol (E6) were purchased and used to synthesize bioreducible and hydrolytically degradable PBAEs (*25*). Either BR6 or B4 was reacted overnight with S4 at 90°C with stirring to form the acrylate-terminated base polymer, which was then end-capped with E6 to form the polymers R646 and 446. siRNA-encapsulating nanoparticles were formed by dissolving siRNA and polymer at the desired concentrations in 25 mM sodium acetate solution (NaAc; pH 5) and mixing the two solutions at a 1:1 volume ratio. Nanoparticles were allowed to self-assemble for 10 min at RT, at which time they were mixed at a 1:1 volume ratio with sodium bicarbonate solution at a final concentration of 9 mg/ml (NaHCO_3_; pH 9). For in vitro transfections, nanoparticle solutions were added directly to cell culture media and incubated for 4 hours. Nanoparticles were formulated at a R646:446 w/w ratio of 9:1 and a polymer: siRNA w/w ratio of 125; the final siRNA dose was 100 nM per well. Characterization data for the nanoparticles are shown in fig. S3.

For in vivo intravitreal injections, nanoparticles were synthesized at a polymer concentration of 5 mg/ml to enable a higher dose to be delivered in the limited injection volume. Nanoparticles were lyophilized in the presence of sucrose (30 mg/ml) as a cryoprotectant and resuspended using deionized water to a final isotonic sucrose concentration of 100 mg/ml immediately before injection.

### Intraocular injections

Intravitreal injections were performed with a Harvard pump microinjection apparatus (South Natick, MA) using pulled-glass micropipettes. Each micropipette was calibrated to deliver a 1-μl volume on depression of a foot switch. The mice were anesthetized, and under a dissecting microscope, the sharpened tip of the micropipette was passed through the sclera just posterior to the limbus into the vitreous cavity, and the foot switch was depressed, which caused fluid to penetrate into the vitreous space.

### Oral gavage

Oral gavage with PT2385 (30 mg/kg) was performed daily for five consecutive days in the OIR mice. PT2385 was dissolved in dimethyl sulfoxide (20 mM), diluted in 0.5% methylcellulose, 0.5% Tween 80, and 0.1% NaCl in water (w/v), and administered daily to C57BL/6 mice from P12 to P16. The dosing volume was 10 ml/kg.

### Patient samples

Institutional Review Board approval from the Johns Hopkins University School of Medicine was obtained for all patient samples used here. Aqueous samples were collected from consenting patients undergoing cataract or vitrectomy surgery.

### Autopsy eyes

Institutional Review Board approval from the Johns Hopkins University School of Medicine was obtained for all autopsy eyes used in this study. Three eyes from three patients with PDR were included for IHC.

### Statistical analysis

Results from cell culture and animal models are shown as means ± SD from at least three independent experiments. Results from clinical samples are shown as means ± SD. Western blot scans are representative of at least three independent experiments. Statistical analysis was performed with Microsoft Excel and Prism 8.0 software (GraphPad). To calculate statistical significance, two-tailed Student’s *t* test, one-way analysis of variance (ANOVA), or two-way ANOVA followed by Bonferroni’s multiple comparisons test was used, as indicated in the figure legends. *P < 0.05; **P < 0.01; ***P < 0.001; ****P < 0.0001. NS, not significant.

## Notes

View/request a protocol for this paper from *Bio-protocol*.
