# Supplementary material for: PAI-1 is a vascular cell–specific HIF-2–dependent angiogenic factor that promotes retinal neovascularization in diabetic patients
Source: Sci Adv. 2022 Mar 2;8(9):eabm1896. doi: 10.1126/sciadv.abm1896 (PMC8890718; doi:10.1126/sciadv.abm1896)
Supplement: Supplementary file 1 — Tables S1 to S3 Figs. S1 to S3 [file sciadv.abm1896_sm.pdf]

Science Advances
AAAS

Supplementary Materials for

PAI-1 is a vascular cell–specific HIF-2–dependent angiogenic factor that promotes retinal neovascularization in diabetic patients

Yaowu Qin, Jing Zhang, Savalan Babapoor-Farrokhran, Brooks Applewhite, Monika Deshpande, Haley Megarity, Miguel Flores-Bellver, Silvia Aparicio-Domingo, Tao Ma, Yuan Rui, Stephany Y. Tzeng, Jordan J. Green, M. Valeria Canto-Soler, Silvia Montaner, Akrit Sodhi*

*Corresponding author. Email: asodhi1@jhmi.edu

Published 2 March 2022, *Sci. Adv.* **8**, eabm1896 (2022)
DOI: 10.1126/sciadv.abm1896

This PDF file includes:

Tables S1 to S3
Figs. S1 to S3

Supplemental Table 1. Characteristics of patients from 3 cohorts for multiplex angiogenesis arrays.

Cohort	Patient Eyes	Age (yr)	Sex	Type of DM	VEGF Levels (ng/ml)	Tubule Formation (Fold Induction vs. mean controls)
PDR Low VEGF	Patient Eye 1	55	M	2	0.09	1.5
	Patient Eye 2	51	F	2	0.13	2.79
	Patient Eye 3	43	M	1	0.07	2.08
	Patient Eye 4	58	M	2	0.16	1.72
PDR Anti-VEGF	Patient Eye 5	49	M	2	0.12	1.42
	Patient Eye 6	35	M	1	0.35	1.3
	Patient Eye 7	33	F	1	0.11	1.25
	Patient Eye 8	33	F	1	0.14	2.1
Controls	Patient Eye 9	71	F	-	1.1	-
	Patient Eye 10	71	F	-	1.1	-
	Patient Eye 11	64	F	-	1.2	-
	Patient Eye 12	49	F	-	0.72	-

Abbreviation: PDR, proliferative diabetic retinopathy; VEGF, vascular endothelial growth factor; DM, diabetes mellitus; yr, years; F, female; M, male.

Supplemental Table 2. Antibodies used in this study.

Name	Company	Host Species	Catalog#	dilution	Marker/Applications
PAI-1	BD Transduction Laboratories™	mouse	BD612025	1:400-1:1000	IF
PAI-1	Santa Cruz	rabbit	SC8979	1:500-1:1000	IF
HIF-1α	Thermo Fisher	rabbit	PA1-16601	1:1000	IF
HIF-1α	Gene Tex	rabbit	GTX127309	1:200-1:1000	IF
HIF-2 α	Novus Biologi	rabbit	NB-100-122	1:200-1:500	IF
Isolectin GS-IB4	Thermo Fisher		I21413	1:200-1:500	IF
CD31	R&D	goat	AF3628	1:400-1:1000	IF
CD34	abcam	rabbit	ab81289	1:100-1:500	IF
NG2	Millipore Sigma	rabbit	AB5320	1:500	IF
GFAP	abcam	goat	AB53554	1:500-1:1000	IF
RBPMS	abcam	rabbit	AB152101	1:200	IF
Isolectin GS-IB4	Invitrogen	Alexa Fluor™ 488 Conjugate	121413	1:200	IHC
PAX-6	DSHB	mouse	AB_528427	1:50	IF
REC	MilliporeSigma	rabbit	AB5585	1:500	IF
CRALBP	abcam	mouse	ab15051	1:500	IF
HIF-2α	R&D	goat	AF2997	1:200	IF
HIF-2α	Novus Biologi	rabbit	NB-100-122	1:200	IHC
PAI-1	Santa Cruz	rabbit	SC8979	1:400	IHC
IgG	Santa Cruz				
secondary antibodies	Invitrogen			1:1000	IF
secondary antibodies	Dako			1:100	IHC

Supplemental Table 3. Primer sequences for Real-Time (RT)-PCR

	Gene		Sequence (5'to 3')
Mouse			
	PAI-1	Forward	TGATGGCTCAGAGCAACAAG
		Reverse	GCCAGGGTTGCACTAAACAT
	Cyclophilin A	Forward	AGCATACAGGTCCTGGCATC
		Reverse	TTCACCTTCCCAAAGACCAC
Human			
	PAI-1	Forward	AGCTCCTTGTACAGATGCCG
		Reverse	ACAACAGGAGGAGAAACCCA
	VEGF	Forward	GGGCAGAATCATCACGAAGT
		Reverse	TGGTGATGTTGGACTCCTCA
	KDR	Forward	CAACCTTCTAGGTGCCTGTAC
		Reverse	GGATATTTCGTCCCGCCTGG
	β-actin	Forward	CTCTTCCAGCCTTCCTTCCT
		Reverse	AGCACTGTGTTGGCGTACAG

Supplemental Figure 1

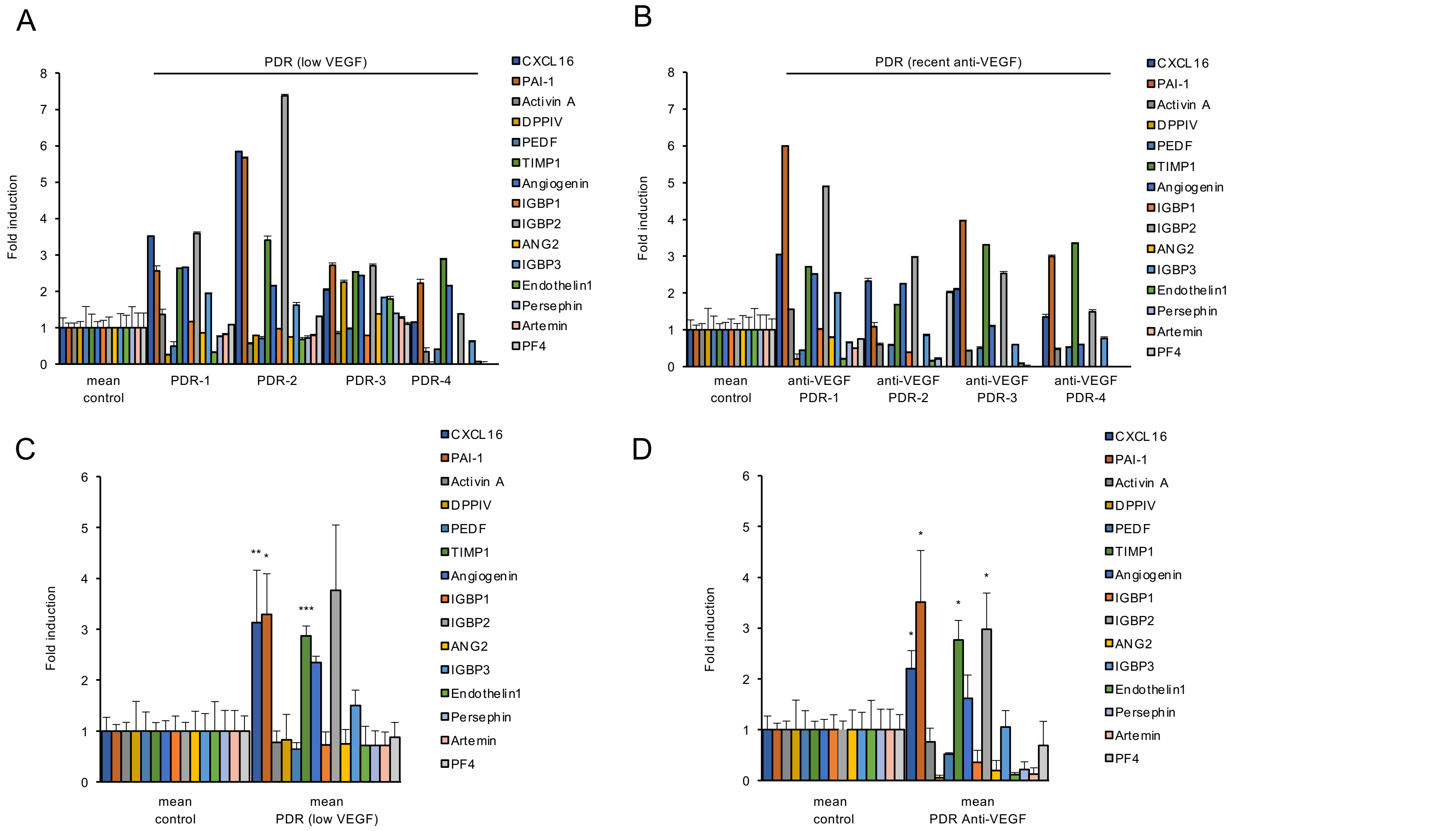

Supplemental Figure 2

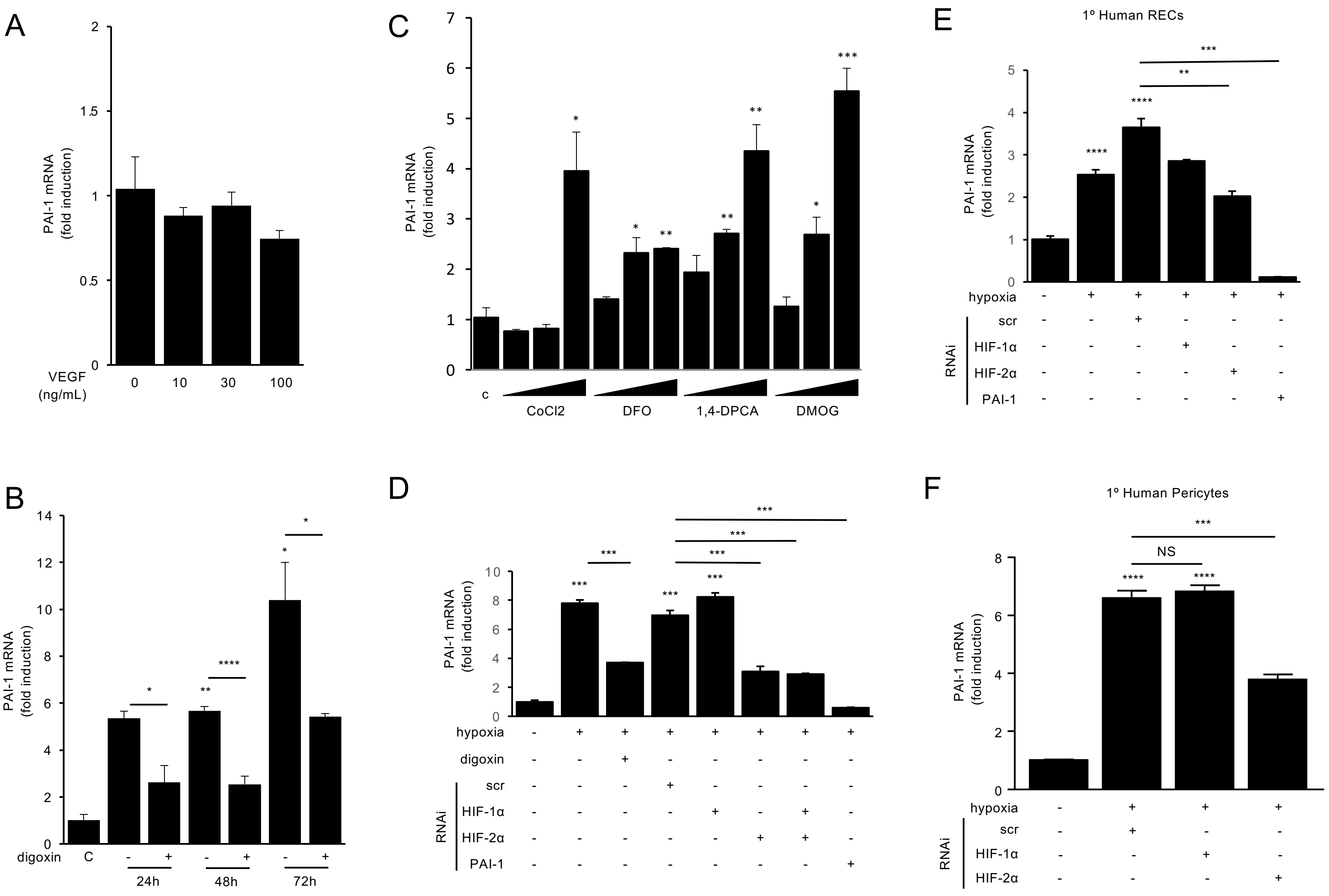

Supplemental Figure 3

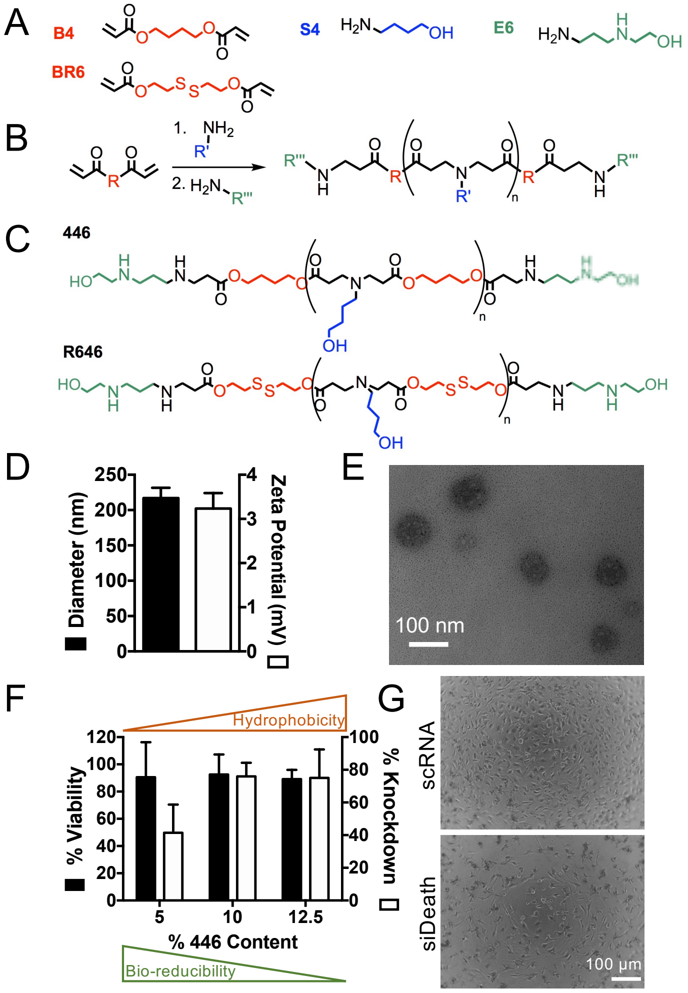

Supplementary Materials

Supplemental Figure 1. PAI-1 expression is increased in PDR patients with low VEGF levels.

(**A-B**) The expression levels of angiogenic cytokines, growth factors, and inflammatory mediators by multiplex ELISA angiogenesis arrays on aqueous fluid (AF) from 4 PDR patients with PRP (**A**), or 4 PDR patients with anti-VEGF (**B**) therapy. (**C-D**) Quantitation of angiogenic mediators on AF from a subset of PDR patients with PRP (**C**), or PDR patients with anti-VEGF (**D**) therapy compared with non-diabetic control patients. Data are shown as mean ± SD. Statistical analyses were performed by two-way ANOVA with Bonferroni's multiple-comparison test. *P < 0.05; **P < 0.01; ***P < 0.001; ****P < 0.0001.

Supplemental Figure 2. HIF-2α, but not HIF-1α, regulates PAI-1 expression in vascular cells *in vitro*.

(**A**) *PAI1* mRNA expression in immortalized human umbilical vein endothelial cell line (iHUVEC) treated with recombinant human (rh) VEGF (at indicated doses). (B) *PAI1* mRNA expression in iHUVEC treated with increased duration of hypoxia in the presence and absence of digoxin. (**C**) *PAI1* mRNA expression in iHUVEC treated with increasing doses of either $CoCl_2$, DFO, 1, 4-DPCA or DMOG. (**D**) The *PAI1* mRNA expression in iHUVEC exposed to hypoxia after treatment with digoxin or knockdown of PAI-1, HIF-1α, HIF-2α or both by RNAi. (**E**) The *PAI1* mRNA expression in primary human retinal endothelial cells (1° Human RECs) exposed to hypoxia after knockdown of PAI-1, HIF-1α, HIF-2α or both by RNAi. (**F**) The *PAI1* mRNA expression in primary human pericytes (1° Human Pericytes) exposed to hypoxia after knockdown of HIF-1α, HIF-2α, or both by RNAi. Data are shown as mean ± SD. Statistical

analyses were performed by one-way ANOVA with Bonferroni's multiple-comparison test (**A, D-F**) or two-way ANOVA with Bonferroni's multiple-comparison test (**B** and **C**). *$P < 0.05$; **$P < 0.01$; ***$P < 0.001$; ****$P < 0.0001$; NS, non-significant.

Supplemental Figure 3. Synthesis, characterization, and *in vitro* evaluation of PBAE-siRNA nanoparticles.

(**A**) Monomer structures. **(B)** Reaction scheme for reducible and hydrolytically degradable poly(beta-amino ester)s (PBAEs). (1) Acrylate-terminated PBAEs are synthesized via Michael addition of B and S monomers; (2) polymer end-capping with monomer E6 yields final polymer products. **(C)** Structures of polymers 446 and R646. **(D)** Nanoparticle hydrodynamic diameter as measured by DLS and zeta potential as measured by electrophoretic mobility on the same instrument. Data represented as mean + SEM, n=3. **(E)** Representative TEM image of nanoparticles. **(F)** Nanoparticle-mediated knockdown in HUVEC cells *in vitro* using combination R646 and 446 nanoparticles. Knockdown was assessed by delivering death positive control siRNA; functional siRNA knockdown results in cell death. Data represented as mean + SEM, n=4. **(G)** Microscopy images of HUVEC cells treated with nanoparticles (90% R646/10% 446) delivering 100 nM siRNA. scRNA = scrambled control RNA; siDeath = positive control death mix siRNA.
